# Phoenixin Expression Is Regulated by the Fatty Acids Palmitate, Docosahexaenoic Acid and Oleate, and the Endocrine Disrupting Chemical Bisphenol A in Immortalized Hypothalamic Neurons

**DOI:** 10.3389/fnins.2018.00838

**Published:** 2018-11-15

**Authors:** Emma K. McIlwraith, Neruja Loganathan, Denise D. Belsham

**Affiliations:** ^1^Department of Physiology, University of Toronto, Toronto, ON, Canada; ^2^Department of Obstetrics and Gynaecology, University of Toronto, Toronto, ON, Canada; ^3^Department of Medicine, University of Toronto, Toronto, ON, Canada

**Keywords:** phoenixin, hypothalamus, fatty acids, endocrine-disrupting chemical bisphenol A, gene expression, signal transduction

## Abstract

Phoenixin (PNX) is a newly identified reproductive peptide required for the estrous cycle. It is most highly expressed in the hypothalamus, where it is a positive regulator of gonadotropin-releasing hormone (GnRH) and kisspeptin. However, it is unknown what signals lie upstream of *Pnx* to coordinate its effects on GnRH and kisspeptin. We investigated the effects of the hormones, estrogen and leptin; the fatty acids, palmitate, docosahexaenoic acid (DHA), oleate and palmitoleate; and the endocrine disrupting chemical BPA on *Pnx* mRNA levels. We also examined whether the signaling pathways of nitric oxide, lipopolysaccharide, cAMP and protein kinase C could alter *Pnx* expression. Immortalized hypothalamic neurons were treated from 2 to 24 h with these compounds and *Pnx* mRNA levels were measured with RT-qPCR. Unexpectedly, only BPA as well as the fatty acids, palmitate, DHA and oleate, could alter *Pnx* expression; therefore suggesting that *Pnx* may fulfill a nutrient-sensing role in the hypothalamus. Our study is the first to delineate potential regulators of this novel neuropeptide, and our findings provide some insight into the functional role of PNX in the hypothalamus.

## Introduction

Reproductive function is coordinated by the release of gonadotropin-releasing hormone (GnRH) from the hypothalamus, which stimulates the release of luteinizing hormone (LH) and follicle-stimulating hormone (FSH) from the pituitary, which in turn trigger release of steroid hormones from the gonads (Clarke and Cummins, 1982; Radovick et al., 1991). The steroid hormones provide feedback to the hypothalamus and pituitary, thereby forming the hypothalamic-pituitary-gonadal (HPG) axis. While GnRH is critical to control puberty and the estrous cycle (Barbieri, 2014; Plant, 2015), it is becoming increasingly clear that other peptides contribute to the neural control of reproduction. For example, kisspeptin, a now well-studied peptide, acts as an afferent regulator of GnRH, controlling pulsatile GnRH secretion as well as the preovulatory GnRH surge (Gottsch et al., 2006; Clarkson et al., 2008). Extensive research on kisspeptin has led to its use in clinical trials for *in vitro* fertilization, with promising results (Abbara et al., 2015; Prague and Dhillo, 2015). Input from other peptides, such as gonadotropin-inhibitory hormone (GnIH) and neuropeptide Y (NPY), to GnRH neurons is also involved in normal reproductive function (Dhillon et al., 2009; Roa and Herbison, 2012; Ubuka et al., 2013).

One newly identified peptide crucial for reproductive function is phoenixin (PNX) (Yosten et al., 2013). It is conserved amongst humans, rodents, bovine, pigs, and gallus (Yosten et al., 2013). PNX is cleaved from small integral membrane protein 20 (SMIM20) into an amidated 14 or 20 amino acid chain, referred to as PNX-14 and PNX-20, respectively (Yosten et al., 2013). Yosten et al. (2013) showed siRNA knockdown of the peptide extended the rat estrous cycle by an average of 2.3 days, demonstrating it is indispensable for normal estrous cycling (Yosten et al., 2013). This effect may occur through its actions at two levels of the HPG axis: the hypothalamus and pituitary. In the hypothalamus, where PNX is most highly expressed, PNX positively regulates GnRH expression and secretion, as well as kisspeptin expression (Treen et al., 2016). In the pituitary, PNX enhances GnRH-stimulated LH release (Yosten et al., 2013; Stein et al., 2016). These actions are mediated by the PNX receptor, GPR173, which is a conserved G protein coupled receptor, expressed highly in the brain and ovaries (Matsumoto et al., 2000; Stein et al., 2016; Treen et al., 2016). However, how this positive regulator of the HPG axis is itself controlled remains unknown.

Determining the signals that stimulate PNX to ultimately alter GnRH and kisspeptin is critical to understanding the role of PNX in the hypothalamus. The expression of *Pnx* at the hypothalamic level may allow it to be modulated by peripheral signals, including hormones and fatty acids, that cross the blood–brain barrier at the median eminence and are known to regulate hypothalamic peptide expression. We took two approaches to determine such regulators of *Pnx* expression. The first was, as mentioned, to determine *Pnx* gene expression after exposure to hormones and fatty acids. Given that *Pnx* is involved in the HPG axis, it may be regulated by estrogen, which provides feedback to the axis (Barraclough and Haller, 1970; Radovick et al., 1991). Another hormone, leptin, is secreted from adipose tissue and is thought to signal the HPG axis as a measure of nutritional status (Barash et al., 1996; Perez-Perez et al., 2015). Furthermore, it is possible that *Pnx* would be affected by hormone mimics, such as the endocrine disrupting chemical, bisphenol A (BPA), which is thought to have estrogenic activity (Acconcia et al., 2015). Additionally, *Pnx* may be affected by nutritional signals like fatty acids, which can regulate expression of the reproductive peptides, GnRH and LH (Garrel et al., 2011; Tran et al., 2016). Our second approach was to identify common signaling pathways that potentially regulate *Pnx* in an attempt to identify other regulatory pathways controlling *Pnx* expression. Such signaling molecules include cAMP, protein kinase C (PKC), nitric oxide (NO) and lipopolysaccharide (LPS).

The hypothalamus contains a heterogeneous array of neurons that are modulated differentially by hormones and fatty acids. Our laboratory has generated immortalized hypothalamic cell lines to study the responses of individual neuronal populations (Belsham et al., 2004). Therefore, to study the regulation of *Pnx* in the hypothalamus, we used the immortalized clonal *Pnx*-expressing mHypoE-46 cell line, as it has been used on numerous occasions to study the effects of leptin, insulin and palmitate in the hypothalamus (Mayer and Belsham, 2009; Dhillon and Belsham, 2011; Ye et al., 2016). We also used cell lines representing male, female, adult and embryonic neurons, allowing for the study of sex and developmental differences. Here, we demonstrate that the fatty acids palmitate, docosahexaenoic acid (DHA) and oleate, as well as BPA modulate *Pnx* expression in immortalized hypothalamic cell models. We also demonstrate that 17-ß estradiol (E2), leptin, palmitoleate, cAMP, NO, PKC and LPS do not regulate *Pnx* gene expression. Together, these results provide the first evidence of compounds that can and cannot regulate the newly identified peptide, PNX, in the hypothalamus.

## Materials and Methods

### Cell Culture

Clonal hypothalamic cell lines were immortalized using SV40 T-antigen, as previously described (Belsham et al., 2004, 2009). These cell lines express *Pnx* and *Gpr173*, along with receptors and neuropeptides, and represent neurons from adult, embryonic, male and female mice (Table 1). The effects of all compounds were tested in the embryonic male cell line, mHypoE-46. Given that estrogen has differential effects during development and between sexes, E2 and BPA, which has estrogenic activity, were also studied in the other cell lines.

**Table 1:** Immortalized hypothalamic cell line characteristics.

Cell line	*Pnx*	*Gpr173*	*Esr1*	*Esr2*	*Gpr30*	*Agrp*	*Histone 3a*
mHypoE-46 (M)	+++	++	++	++	+++	++	++++
mHypoA-2/12 (M)	+++	++	++	+	++	++	++++
mHypoE-41 (F)	+++	++	++	++	++	++	++++
mHypoA-59 (F)	+++	++	++	++	++	++	++++

*The cell lines express Pnx and its receptor, Gpr173, as well as other receptors and neuropeptides, for example, the estrogen receptors Esr1, Esr2, and Gpr30, and agouti-related peptide (Agrp). Gene expression was assessed by RT-qPCR and the relative expression is represented by the ranges: ++++CT < 20.0, +++20.0 < CT < 25.0, ++ 25.0 < CT < 30.0, + 30.0 < CT < 35.0, - CT > 35.0. The “E” or “A” within the name denotes they are derived from embryonic or adult mice, respectively, and (M) or (F) denotes male or female.*

Cells to be treated with E2 or BPA were cultured in Dulbecco’s Modified Eagle Medium (DMEM) with 25 mM glucose (Sigma-Aldrich), supplemented with 2% fetal bovine serum (FBS; Gibco, Life Technologies) and 1% penicillin/streptomycin (P/S; Gibco, Life Technologies). To eliminate influence of steroids contained in DMEM and FBS, treatments with E2 and BPA were performed in phenol-red free DMEM (Hyclone, Fisher Scientific), supplemented with 1% charcoal:dextran-stripped FBS (Gemini Bio Products through Cedarlane, Inc.) and 1% P/S. Cells to be treated with all other compounds were grown and treated in 5.5 mM glucose DMEM, supplemented with 2% FBS and 1% P/S.

### Primary Culture

Eight-week-old CD1 female or male mice were euthanized with CO_2_ and their hypothalamic extracted. This study was carried out in accordance with the recommendations of the Ontario’s Animals for Research Act, and the federal Canadian Council on Animal Care. All animal procedures were approved by the University of Toronto animal care committee. Cells were grown in Neurobasal A medium (Gibco, Life Technologies) containing 1 × GlutaMAX supplement (Gibco), 1 × B27 serum-free supplement (Gibco), 10% FBS, 5% normal horse serum, and 1% P/S (Gibco, Life Technologies) for 1 week, with 10 ng/μL ciliary neurotrophic factor (CNTF) addition each day. Primary culture was treated with 100 μM BPA for 8 h or 50 μM palmitate for 16 h in DMEM as described below.

### Reagents

E2 (Tocris Bioscience) was dissolved in 100% ethanol and diluted to 10 or 100 μM in sterile-filtered Hypure (Hyclone, Fisher Scientific) or UltraPure (Thermofisher Scientific) water followed by a 1:1,000 dilution in culture media to a final concentration of 10 or 100 nM in 0.0005% ethanol. 200 mM BPA (Sigma-Aldrich) was dissolved in 100% ethanol and diluted to 100 mM with sterile-filtered water; a 1:1,000 dilution was then performed in culture media to obtain a final concentration of 100 μM BPA containing 0.05% ethanol. Sodium palmitate and sodium oleate (Sigma-Aldrich) were dissolved by heating to 60°C in water, and added to media at 50 μM. Sodium nitroprusside (SNP) (Sigma-Aldrich) was dissolved in sterile-filtered water and added to media to obtain 100 μM SNP.

All stock solutions were diluted 1:1,000 in culture media to obtain the final concentration. Palmitoleate (Sigma-Aldrich) 200 mM stock solution was prepared in 50% ethanol and by heating to 60°C. Immediately before use, palmitoleate was diluted to 100 mM in 50% ethanol and 100 μM in media. DHA (Sigma-Aldrich) was dissolved in DMSO to obtain a 100 mM stock solution, followed by dilution to 100 μM in media. 100 μM leptin (National Hormone and Peptide Program) stock solution was prepared in 1× PBS and diluted to 100 nM in media. 100 μg/mL LPS (O55:B5; Sigma-Aldrich) stock solution was prepared in sterile-filtered water, and diluted to 100 ng/mL in media. 10 mM Forskolin and 100 μM TPA (Sigma-Aldrich) stock solutions were prepared in DMSO, then diluted to 10 μM and 100 nM in media, respectively. Cells were grown to 70–80% confluency for treatment.

### Quantitative Real-Time PCR

Total RNA was isolated using the PureLink RNA Mini Kit (Ambion, Life Technologies) and its purity and concentration were quantified using the Nanodrop 2000c spectrophotometer. cDNA was synthesized with the High Capacity cDNA Reverse Transcription Kit (Applied Biosystems). For qRT-PCR, 12.5 ng of cDNA was amplified with gene-specific primers and Platinum SYBR Green qPCR SuperMix-UDG with ROX (Life Technologies, Thermofisher Scientific) in the Applied Biosystems 7900 HT Real-Time PCR machine. The primers are listed in Table 2 and were designed using the online tool, PrimerBLAST (Ye et al., 2012). Data was analyzed using the standard curve method and normalized to the standardization gene, *Histone 3A*, except BPA-treated samples which were normalized to *Rpl7*.

**Table 2:** List of primer sequences.

Gene	Primer sequence (5′→3′)	Amplicon size (bp)
*Histone 3a*	F: CGC TTC CAG AGT GCA GCT ATT	72
	R: ATC TTC AAA AAG GCC AAC CAG AT	
*Rpl7*	F: TCG CAG AGT TGA AGG TGA AG	114
	R: GCC TGT ACT CCT TGT GAT AGT G	
*Pnx*	F: AGC AAG CTG TAA ATC GAG CTG GTA	146
	R: ACT GCG GAG TGC ACA GGA TAA AGA	
*Gpr173*	F: CTG GCG AGT GTT TGT GAA AG	125
	R: TCT TGA GGT CCT TGT TAA GCA	
*Esr1*	F: GAG TGC CAG GCT TTG GGG ACT T	102
	R: CCA TGG AGC GCC AGA CGA GA	
*Esr2*	F: ATC TGT CCA GCC ACG AAT CAG TGT	114
	R: TCT CCT GGA TCC ACA CTT GAC CAT	
*Gpr30*	F: AGCTGATGTTCACCACCAGGATGA	104
	R: TCAGCAGTACGTGATTGCCCTCTT	
*Agrp*	F: CGG AGG TCG TAG ATC CAC AGA	69
	R: AGG ACT CGT GCA GCC TTA CAC	
*Npy*	F: CAG AAA ACG CCC CCA GAA	77
	R: AAA AGT CGG GAG AAC AAG TTT CAT	
*Stat3*	F: GCC ACG TTG GTG TTT CAT AAT C	97
	R: TTC GAA GGT TGT GCT GAT AGA G	
*Il-6*	F: GTG GCT AAG GAC CAA GAC CA	85
	R: GGT TTG CCG AGT AGA CCT CA	
*iNos*	F: CCT GAA GGT GTG GTT GAG TT	124
	R: CTT GGA AGA GGA GCA ACT ACT G	
*Bmal1*	F: GGG AGG CCC ACA GTC AGA TT	78
	R: GTA CCA AAG AAG CCA ATT CAT CAA	

### Statistical Analysis

Results were expressed as mean ± SEM and analyzed with GraphPad Prism Software 6.0 (GraphPad Software, Inc.). Statistical significance was determined with a two-way ANOVA or a *T*-test, as appropriate, followed by a Bonferroni *post hoc* test, where ^∗^*P* < 0.05, ^∗∗^*P* < 0.01, ^∗∗∗^*P* < 0.001 and ^∗∗∗∗^*P* < 0.0001.

## Results

### E2 Does Not Alter *Pnx* mRNA Levels in Immortalized Hypothalamic Neurons

**FIGURE 1:**
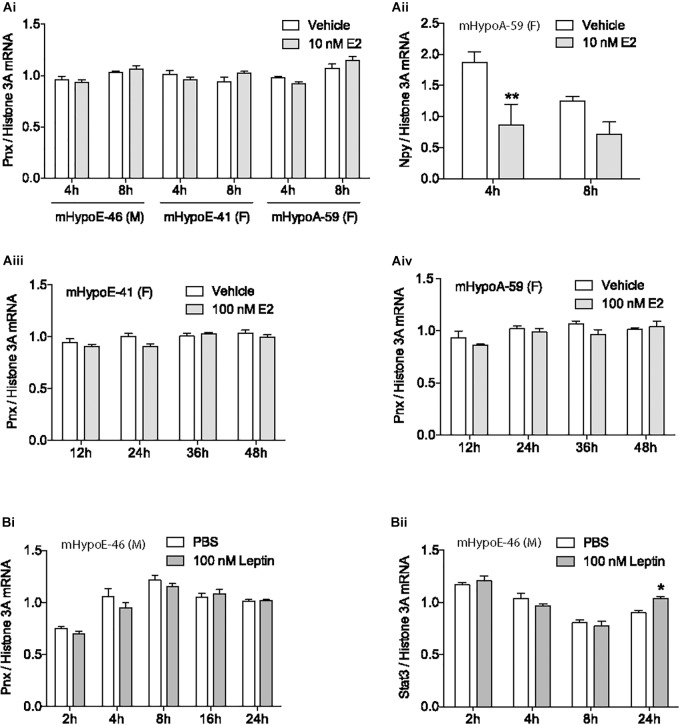
Estrogen and leptin do not alter *Pnx* gene expression. **(Ai)** Treatment with 10 nM of 17β-estradiol (E2) for 4 or 8 h in the mHypoE-46, mHypoE-41 and mHypoA-59 cell lines (*n* = 4). **(Aii)** *Npy* expression after treatment with 10 nM E2 in the mHypoA-59 cell line (*n* = 4). **(Aiii)** Treatment with 100 nM E2 for 12, 24, 36, and 48 h in the mHypoE-41 cell line. **(Aiv)** Treatment with 100 nM E2 for 12–48 h in the mHypoA-59 cell line. **(Bi)** Treatment over 24 h with 100 nM leptin in the mHypoE-46 cell line (*n* = 3). **(Bii)** *Stat3* expression after treatment with 100 nM leptin in the mHypoE-46 cell line (*n* = 4) (mean ± SEM, ^∗^*P* < 0.05 and ^∗∗^*P* < 0.01, by two-way ANOVA).

Mounting evidence demonstrates that the newly identified peptide, PNX, is integral to the HPG axis at the level of the hypothalamus (Yosten et al., 2013; Stein et al., 2016; Treen et al., 2016), but precisely how it is involved in the HPG axis is unknown. To define its role, we examined how the gonadal hormone E2, which is vital to the HPG axis in both females and males (Radovick et al., 1991; Hayes et al., 2000), affects *Pnx* gene expression. The mHypoE-46, mHypoE-41 and mHypoA-59 immortalized clonal hypothalamic neuronal cell lines were treated with E2 and the mRNA levels of *Pnx* were measured with RT-qPCR. In addition to estrogen receptors, these cell lines express both the *Pnx* gene and receptor, similar to GnRH neurons, which co-express the GnRH receptor (Cheng and Leung, 2005), making them appropriate models for analyzing changes in gene expression in response to E2. 10 nM E2 treatment for 4 or 8 h in the male mHypoE-46 cell line, and female mHypoE-41 and mHypoA-59 cell lines had no significant effect on mRNA levels of *Pnx* (Figure 1Ai). E2 treatment did, however, change *Npy* mRNA levels in the mHypoA-59 cell line, as previously reported (Titolo et al., 2006) (Figure 1Aii), demonstrating the responsiveness of these neurons to E2. Further, longer E2 treatment from 12 to 48 h did not alter *Pnx* mRNA levels in the mHypoE-41 (Figure 1Aiii) and mHypoA-59 cell lines (Figure 1Aiv). Therefore, it appears that *Pnx* is expressed independently of E2 feedback in these hypothalamic cell lines.

### Leptin Does Not Alter *Pnx* mRNA Levels

Another hormone with input to the reproductive axis is leptin. Secreted from adipose tissue, leptin signals to the hypothalamus to reduce food intake and increase energy expenditure (Sahu, 2003). Leptin is tightly coupled to reproductive capacity, for example, leptin infusion increases secretion of GnRH and LH in rats (Watanobe, 2002; Hausman et al., 2012). However, treatment with 100 nM leptin over 24 h did not alter *Pnx* expression in the mHypoE-46 cell line (Figure 1Bi), signifying that *Pnx* expression may not be related to adipose stores. In contrast, as a positive control, we found that leptin increased Stat3 mRNA levels at 24 h (Figure 1Bii).

### The Fatty Acids Palmitate, DHA and Oleate, but Not Palmitoleate, Upregulate *Pnx* mRNA

**FIGURE 2:**
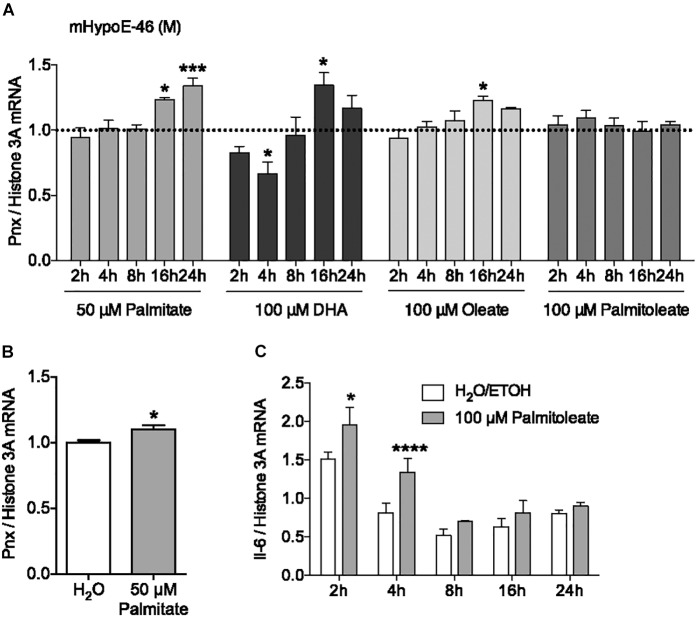
Fatty acids regulate *Pnx* mRNA levels in cell lines and hypothalamic primary culture. **(A)** The mHypoE-46 cell line was treated with palmitate, DHA, oleate and palmitoleate over 24 h (*n* = 4). Bars represent treatments normalized to 1 (dotted line indicates time-matched controls set to 1). **(B)** Male murine primary culture was treated with 50 μM palmitate for 24 h (*n* = 4; normalized to 1). **(C)** *Il-6* mRNA levels after treatment with 100 μM palmitoleate in the mHypoE-46 cell line (*n* = 4) (mean ± SEM, ^∗^*P* < 0.05, ^∗∗∗^*P* < 0.001, and ^∗∗∗∗^*P* < 0.0001, by **(A,C)** two-way ANOVA or **(B)** *T*-test).

In addition to hormonal input, the HPG axis receives feedback from nutritional signals. It has been reported that hypothalamic neurons can directly sense nutrients, such as free fatty acids, from the blood (Migrenne et al., 2006; Jo et al., 2009). To investigate the effect of the saturated fatty acid palmitate on *Pnx* expression, its mRNA levels were measured over 24 h (Figure 2A). 50 μM palmitate significantly increased *Pnx* expression in the male mHypoE-46 cell line. To determine whether this *Pnx* increase in this male clonal cell line was representative of the whole hypothalamus, we treated male hypothalamic primary culture with 50 μM palmitate for 24 h (Figure 2B). An increase in *Pnx* was observed, suggesting *Pnx* is responsive to palmitate across an array of neuronal populations.

To determine if unsaturated fatty acids could also affect *Pnx* mRNA, the effects of the monounsaturated fatty acids, oleate and palmitoleate, and the polyunsaturated fatty acid, DHA, were measured from 2 to 24 h in the mHypoE-46 neurons (Figure 2A). 100 μM oleate and DHA increased *Pnx* expression, while palmitoleate did not significantly alter *Pnx*. The expression of the cytokine, *interleukin-6 (Il-6)*, was altered in response to palmitoleate, demonstrating neuronal response to the palmitoleate (Figure 2C). Together, this suggests that *Pnx* is responsive to multiple fatty acids.

### BPA Reduces *Pnx* Expression in Male Immortalized and Primary Hypothalamic Cell Culture

**FIGURE 3:**
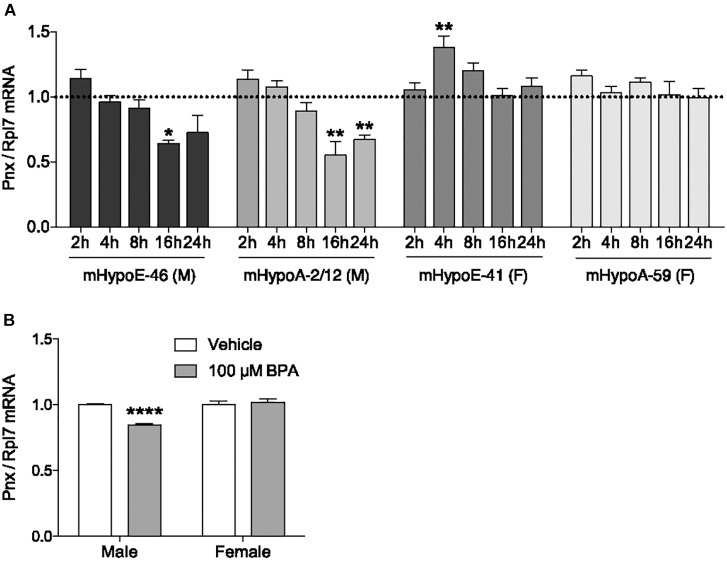
Bisphenol A reduces *Pnx* expression in male cell lines and primary culture. **(A)** Treatment with 100 μM BPA for 2, 4, 8, 16, and 24 h in the mHypoE-46, mHypoA-2/12, mHypoE-41 and mHypoA-59 cell lines (*n* = 4). Bars represent treatment normalized to 1 (dotted line indicates time-matched controls set to 1). **(B)** Male and female primary culture treated with 100 μM BPA for 8 h (*n* = 7; normalized to 1) (mean ± SEM, ^∗^*P* < 0.05, ^∗∗^*P* < 0.01, ^∗∗∗^*P* < 0.001, ^∗∗∗∗^*P* < 0.0001, by **(A)** two-way ANOVA or **(B)** *T*-test).

In addition to determining what physiological compounds could regulate *Pnx*, we assessed whether compounds could dysregulate *Pnx* in the hypothalamus. One environmental chemical with potential to disrupt *Pnx* is BPA, due to its association with reproductive dysfunction such as impairing embryo implantation and reducing sperm quality (Peretz et al., 2014; Tomza-Marciniak et al., 2018). Exposure to BPA *in utero* or in adult mice demonstrates that it has actions in the hypothalamus, for example, it alters the expression of GnRH and kisspeptin (Bai et al., 2011; Kurian et al., 2015). We therefore assessed whether it also alters *Pnx* mRNA. To determine the effect of BPA on *Pnx* gene expression, mRNA levels were measured after 2, 4, 8, 16 and 24 h of 100 μM BPA treatment (Figure 3A). This dosage has been previously found to be effective in our cell lines with the modulation of pro-opiomelanocortin (POMC) and therefore was used here (Salehi et al., 2018). In the mHypoE-46 embryonic male-derived cell line, *Pnx* expression was downregulated at 16 h. *Pnx* expression was also decreased at 16 and 24 h in the adult male-derived mHypoA-2/12 cell line. *Pnx* expression in the embryonic female-derived mHypoE-41 cell line increased after exposure to 100 μM BPA at 4 h, but was unaffected in the adult female mHypoA-59 cell line. Therefore there are potentially differential responses of *Pnx* to BPA in male-derived and female-derived cell lines.

To investigate how BPA affects *Pnx* in a heterogeneous non-immortalized population of cells, male and female primary hypothalamic culture were treated with BPA. Consistent with the observations in clonal cell lines, 8-h treatment with 100 μM BPA decreased expression of *Pnx* in male primary culture, while having no effect on *Pnx* expression in female primary culture (Figure 3B). Taken together, these results suggest that BPA disrupts production of *Pnx* in the male murine hypothalamus.

### Activation of Inflammation and PKC, and Increasing NO and cAMP Levels Have No Effects on *Pnx* Expression

**FIGURE 4:**
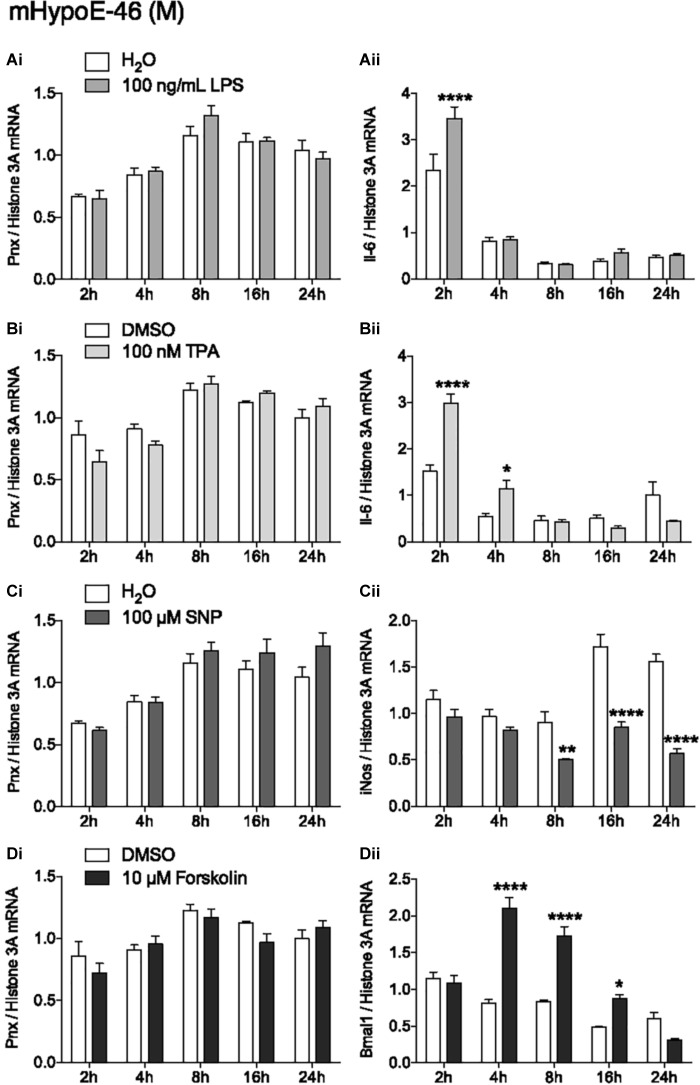
None of the classic signal transduction pathways influence *Pnx* mRNA levels. Treatment over 24 h in the mHypoE-46 cell line with **(Ai,ii)** 100 ng/mL LPS, **(Bi,ii)** 100 nM TPA, **(Ci,ii)** 100 μM SNP and **(Di,ii)** 10 μM forskolin (*n* = 4) (mean ± SEM, ^∗^*P* < 0.05 and ^∗∗∗∗^*P* < 0.0001, by two-way ANOVA).

In addition to identifying hormones and nutrients that regulate *Pnx*, identifying signaling molecules upstream of the *Pnx* gene could also provide information about its regulation. Treatment with LPS to induce the inflammatory signaling pathway did not change *Pnx* expression (Figure 4Ai), but did alter *Il-6* mRNA levels at 2 h, indicating that the inflammatory pathway was activated within the cells (Han et al., 2013) (Figure 4Aii). Activating PKC with 12-*O*-tetradecanoylphorbol-13-acetate (TPA) also did not change *Pnx* mRNA levels (Figure 4Bi), but it did significantly alter *Il-6* expression at 2 and 4 h (Chen et al., 2012) (Figure 4Bii). Increasing NO levels with the NO donor, SNP, also did not alter *Pnx* expression (Figure 4Ci); however, inducible NO synthase (*iNOS*) expression was reduced as a result (Chang et al., 2004) (Figure 4Cii). Induction of cAMP through activation of adenylyl cyclase by forskolin did not alter *Pnx* expression (Figure 4Di), but did alter the positive control *Bmal1* (Chalmers et al., 2008) (Figure 4Dii). Therefore, although the cells were responsive to the signaling molecules tested, it appears that *Pnx* is not regulated by cAMP, NO, PKC or neuroinflammation. Signaling pathways that do regulate *Pnx* remain to be identified.

## Discussion

Phoenixin is a recently identified peptide involved in the HPG axis through positive regulation of GnRH, kisspeptin and LH (Yosten et al., 2013; Treen et al., 2016). However, the regulation of PNX itself was unknown. We investigated whether several hormones, fatty acids and signaling pathways could affect mRNA levels of the *Pnx* gene in the area it is most populous, hypothalamic neurons. Remarkably, only four compounds altered *Pnx* gene expression: palmitate, DHA, oleate and BPA. These findings may point to the specific role of PNX in regulating the reproductive axis.

The fatty acids palmitate, DHA and oleate upregulated *Pnx* expression in the neurons, suggesting a role for PNX in nutritional sensing. Neurons in the hypothalamus, particularly the arcuate nucleus, project to the median eminence where the larger fenestrations allow for the passage of large molecules. In particular, GnRH neurons project directly to the median eminence and have been shown to directly respond to palmitate and DHA *in vitro* (Yin et al., 2009). Without sufficient nutritional resources, it is beneficial for organisms to delay reproduction; therefore *Pnx* production increasing in response to these fatty acids could be a signal to promote reproduction. Indeed, other peptides that respond to metabolic cues have reproductive input. For example, kisspeptin neurons receive input from leptin, ghrelin and insulin (Forbes et al., 2009; Cravo et al., 2013; Qiu et al., 2013), while neurons expressing the feeding neuropeptides, POMC and NPY, project to GnRH neurons (Roa and Herbison, 2012). Furthermore, mice with POMC neurons lacking both the leptin and insulin receptors have reduced fertility (Hill et al., 2010). As *Pnx* appears to be specifically responsive to fatty acids, it may play a larger role in the nutritional control of reproduction than these other peptides that are also responsive to hormonal signals. Alternatively, although PNX is primarily associated with reproductive signaling, it is also related to feeding, therefore its ability to respond to fatty acid signaling may be due to this function. PNX appears to be orexigenic as it modestly increases light phase food intake in rats (Schalla et al., 2017). Furthermore, PNX serum levels increase postprandially (Rocca et al., 2017) and with fasting in the hypothalamus (Wang et al., 2018), suggesting the peptide is responsive to metabolic or hormonal nutritional cues. Therefore, the response of *Pnx* to fatty acids could also be due to its association with satiety and satiation.

The mechanism underlying the effects of palmitate, DHA and oleate may involve multiple signaling pathways. One possibility is that palmitate, DHA and oleate may be acting through the long chain fatty acid receptors, GPR40 and GPR120 (Oh et al., 2010; Nakamoto et al., 2013). Palmitate also activates neuroinflammatory signaling (Shi et al., 2006), however, its effects on *Pnx* mRNA appear to be independent of neuroinflammation as treatment with LPS, which activates toll-like receptor 4, caused no change in expression. Moreover, DHA and oleate, unlike palmitate, have anti-inflammatory actions but also increased *Pnx* (Kwon et al., 2014). Therefore, the effects of fatty acids on *Pnx* appear to be in response to nutritional cues rather than inflammatory cues.

The endocrine disrupting chemical BPA selectively decreased *Pnx* expression in male-derived cell lines and primary culture. This sex difference is not unusual for BPA, as it has also been associated with sex dependent changes in hormones and reproductive functions (Tomza-Marciniak et al., 2018). For example, perinatal exposure to BPA has been noted to alter methylation enzymes and ERα expression differentially in the cortex and hypothalamus of male and female rats, establishing permanent differences in gene expression (Kundakovic et al., 2013). Additionally, neonatal exposure to BPA reduces arcuate kisspeptin fiber density in female, but not male rats (Patisaul et al., 2009).

Bisphenol A may act through multiple mechanisms to alter *Pnx* expression. Although the chemical is a putative estrogen mimic and has been shown to activate nuclear and membrane bound estrogen receptors in a variety of cellular models (Welshons et al., 2006), it appears to be acting through an estrogen-independent pathway in the cell models used in this study, as E2 had no effect on *Pnx* expression. Other mechanisms implicated in the action of BPA include binding and activating the transcription factors estrogen related receptor gamma (ERRγ) or peroxisome proliferator-activated receptor gamma (PPARγ), or activating the inflammatory or endoplasmic reticulum stress pathways (Takayanagi et al., 2006; Asahi et al., 2010; Zhu et al., 2015; Ahmed and Atlas, 2016). BPA is widely known to disrupt the reproductive axis, and our studies provide evidence of yet another way this endocrine disrupting chemical can impact reproduction by altering the expression of a peptide that regulates the HPG axis.

Interestingly, the majority of compounds tested did not alter *Pnx* mRNA levels. E2, a critical component of the HPG axis, did not affect *Pnx* mRNA levels, which was particularly unexpected since PNX is predominantly associated with reproduction and the estrous cycle. Therefore, unlike kisspeptin and GnRH (Roy et al., 1999; Dubois et al., 2014), PNX may influence the HPG axis in an estrogen-independent manner. Instead, based on its response to fatty acids, it may modulate the HPG axis in a nutrient-dependent manner. Investigating other reproductive hormones, such as androgens, for their effects on *Pnx* would be required to further elucidate how PNX is involved in the HPG axis. Leptin, another hormone that did not alter *Pnx*, is secreted from adipose tissue and signals to alter expression of both appetite regulating and reproductive peptides (Barash et al., 1996; Baver et al., 2014). Here, its inability to change *Pnx* may again suggest *Pnx* is more involved with sensing nutrients such as fatty acids, rather than as a sensor of long-term energy stores. Additionally, the general signaling molecules investigated did not alter *Pnx* expression. cAMP and PKC are downstream of the Gαs and Gαq G proteins, respectively. This suggests that compounds that signal through GPCRs coupled to Gαs and Gαq may not affect *Pnx* mRNA levels. These G proteins, however, do activate other pathways, so they may nevertheless affect gene expression and furthermore, they could affect PNX secretion, which was not examined. NO, which can act as an extracellular and intracellular messenger (Tuteja and Chandra, 2004) and has been shown, in immortalized GnRH neurons, to stimulate GnRH secretion (Mahachoklertwattana et al., 1994) did not affect *Pnx*. Additionally, the bacterial endotoxin, LPS, had no effect on *Pnx* mRNA levels, even though it is known to affect other reproductive peptides, such as decreasing GnRH mRNA in ewes (Herman and Tomaszewska-Zaremba, 2010). This enigma of very few compounds regulating *Pnx* may be explained by the fact that it forms part of the mitochondrial chaperone-like protein complex, MITRAC7 (Dennerlein et al., 2015). Overexpression or knock down of MITRAC7 blocks assembly of cytochrome oxidase c. Therefore it is possible that due to the importance of maintaining SMIM20 levels constant, very few compounds can alter its expression at the gene level. The fact that BPA had the strongest effect is further evidence of the detrimental effects of endocrine disrupting chemicals on physiological processes. Overall, this suggests that the role of PNX is highly precise and therefore very few compounds can alter its expression.

An important consideration, and perhaps a limitation to our study, is whether mRNA levels correlate directly to PNX protein levels. We chose to use mRNA analysis due to the increased reproducibility of the data compared to protein analysis that is notoriously difficult to quantify, both through protein arrays and Western/ELISA experiments. The literature is discrepant when it comes to a consensus. There have been numerous studies that indicate that mRNA levels correlate well to protein in steady-state conditions, with a gene-specific lag in protein synthesis (Liu et al., 2016). The tools to assess PNX protein have not yet been sufficiently tested and validated, thus extensive analysis would have to be undertaken. Optimally all of the treatments should be validated at the protein level; however, this is difficult due to the unique lag time of protein expression and it would be prohibitive to analyze the PNX protein over a full time course for each of the 11 compounds tested in this study.

In summary, we provide evidence that BPA and the fatty acids palmitate, DHA and oleate stimulate *Pnx* gene expression, while E2 does not. Elevated levels of cAMP, NO and activation of PKC and neuroinflammation also had no effect on *Pnx* mRNA. The mechanisms by which BPA and palmitate alter *Pnx* expression have yet to be elucidated and will be the subject of further study. Determining how PNX is regulated is necessary for clarifying its physiological role and identifying if it has therapeutic potential. Knowledge of GnRH and kisspeptin has led to treatment of infertility (Suda et al., 1990; Abbara et al., 2015), so it is conceivable that PNX may also lead to novel treatment opportunities.

## Author Contributions

EM and NL performed the experiments, analyzed the data, and wrote the manuscript. DB provided funding, wrote and edited the paper, and provided project guidance.

## Conflict of Interest Statement

The authors declare that the research was conducted in the absence of any commercial or financial relationships that could be construed as a potential conflict of interest.
